# Realizing the potential of routine viral load testing in sub‐Saharan Africa

**DOI:** 10.1002/jia2.25010

**Published:** 2017-11-12

**Authors:** Wafaa M El‐Sadr, Miriam Rabkin, John Nkengasong, Deborah L Birx

**Affiliations:** ^1^ ICAP at Columbia University New York NY USA; ^2^ Africa Centres for Disease Control and Prevention Addis Ababa Ethiopia; ^3^ Office of Global AIDS Coordinator Washington DC USA

**Keywords:** HIV, viral load, sub‐Saharan Africa

## Introduction

The global HIV response has been remarkably successful. More than 19 million persons living with HIV (PLHIV) have accessed life‐saving antiretroviral therapy (ART) 1 and the annual number of HIV‐related deaths and new HIV infections have both plummeted 1. As countries strive to reach the UNAIDS 90:90:90 targets (i.e. for 90% of PLHIV to be aware of their diagnosis, 90% of those who know their diagnosis to receive ART, and 90% of those on ART to have durable viral load suppression 2), new guidelines, tools and implementation strategies are vitally important.

Viral load measurement is a critical tool to assess the impact of HIV treatment efforts, and is now endorsed by the World Health Organization (WHO) as the primary methodology for monitoring response to ART 3. This recommendation is based on research demonstrating that viral suppression is associated with decreased HIV disease progression and mortality among PLHIV, and the prevention of HIV transmission to sexual partners 4, 5. Although stakeholders were initially slow to adopt this WHO recommendation, most funders and national programmes now strongly support scaling up access to routine viral load monitoring 6.

Because viral load measurement is a laboratory assay, it is unfortunately easy to misunderstand the challenge of viral load scale‐up as one for laboratorians only. However, experience with other laboratory assays in resource‐limited settings has shown that it takes far more than assuring availability for a test to realize its potential. For example, even with the availability of testing for early infant HIV diagnosis, multiple challenges have been described in relation to coverage, quality and utilization of results. These include ensuring correct sampling methodologies for dried blood spot samples from HIV‐exposed infants, obtaining specimens at the recommended timeframes, transporting specimens to the laboratory, ensuring tests are done in a timely fashion, enabling providers to promptly access test results and providing results to the infant's caregiver to enable appropriate clinical decision‐making 7. These impediments delay the diagnosis of HIV infection among infants, thus delaying ART initiation in this vulnerable population 8, 9, 10.

**Figure 1:**
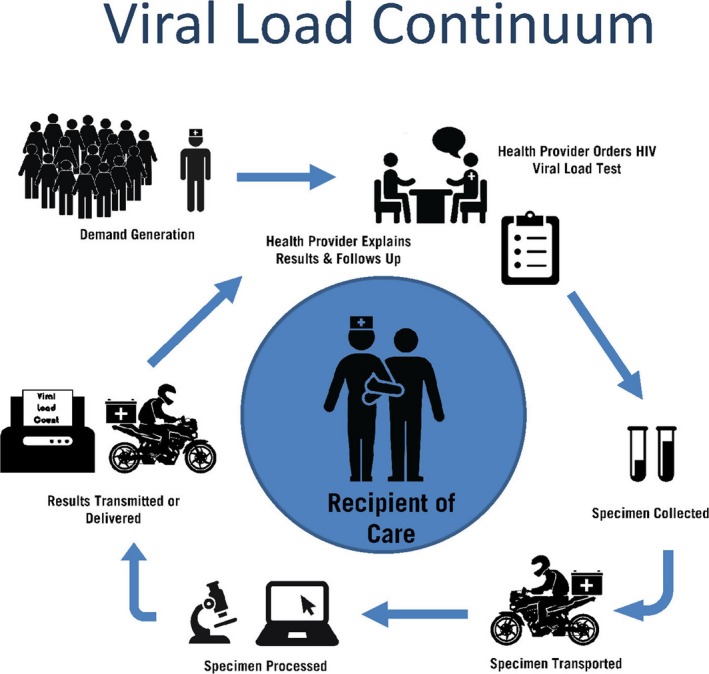
Viral load continuum.

The experience of early infant diagnosis (EID) scale‐up highlights the importance of conceptualizing viral load testing as a continuum; a series of steps, each critical for achieving the ultimate goal – swift and appropriate clinical management to maximize the chance of sustained viral suppression (Figure 1). The first step in the viral load continuum is support for demand generation among both recipients of care and their health providers to motivate the former to request and the latter to order the test.

Once the sample is obtained, transportation systems are required to convey specimens, whether plasma or dried blood spot samples, to the appropriate laboratories. Laboratories need staff, equipment, laboratory information systems and quality assurance/improvement capacity to ensure that specimens are logged, tracked, tested and documented – and that results are transmitted back to the health facility, either electronically or physically. Point‐of‐care assays for viral load measurement offer the potential for overcoming the challenges encountered in transport of specimens and results 11. Nonetheless, irrespective of laboratory method used, these results must then be readily available to the providers, whether in medical charts or via an electronic medical record.

Ultimately, the most critical step is for the providers to review the test results and to share them promptly with the recipients of services. Ensuring that clients are aware of the importance of viral load monitoring and viral suppression for both their own health as well as preventing onward transmission is critically important. Similarly, providers must be trained and supported to swiftly act upon viral load test results, reinforcing clients whose viral load is suppressed, and rapidly and accurately following national guidelines for patients with unsuppressed viral loads to increase adherence or adjust ART regimens if viral resistance is suspected.

In most countries, the first step after an unsuppressed viral load result is to provide enhanced adherence counselling (EAC) based on a careful assessment of all the factors that may be impeding clients’ ability to take ART regularly, followed by repeat viral load measurement 3. In the absence of viral resistance testing – which is unavailable in many of the countries most severely affected by the HIV epidemic – failure to achieve viral suppression after EAC is assumed to reflect viral resistance, motivating change to a second line regimen 12. With the recent expanding availability of integrase inhibitors, e.g. dolutegravir‐containing regimens, simplification of both first and second line treatment is on the immediate horizon with the potential to enhance adherence due to decreased risk of side effects 13. For children living with HIV, it is critically important to accelerate the development of similar regimens, particularly in formulations appropriate for young children 14.

Findings to date indicate that many clients who have unsuppressed viral load achieve viral suppression after EAC, demonstrating that in a significant proportion, the unsuppressed viral load is largely due to non‐adherence 15, 16, 17. This finding is in contrast to a recent report that raised concern regarding an increase in the prevalence of HIV resistance 18. Of note, results from the four recently conducted nationally representative population‐based HIV impact assessments (PHIAs) in Zimbabwe, Malawi, Zambia and Swaziland showed impressive viral load suppression (between 86.5% and 91.9%) among PLHIV who indicated that they were aware of their HIV‐positive status and were on ART, suggesting the robustness of the commonly used current first‐line regimen 19, 20.

This supplement aims to summarize a workshop focused on viral load scale‐up that took place from 27 to 30 June 2016 in Swaziland. The workshop, entitled “Reaching the Third 90: Implementing High Quality Viral Load Monitoring at Scale,” was attended by 150 participants from 16 sub‐Saharan African countries, including individuals from diverse backgrounds reflecting key elements of the viral load continuum 21, such as clinical providers, civil society representatives, laboratorians, programme managers, policy makers, researchers and funders. The workshop agenda was inspired by the concept of the viral load continuum and included cross‐disciplinary panels and small group discussions to encourage attendees to think broadly beyond their own disciplines and areas of interest. The articles included in this supplement reflect this premise.

In their article, Killingo *et al*. describe the efforts of the International Treatment Preparedness Coalition to mobilize communities of PLHIV to demand access to viral load testing and to empower them to advocate for such access in the countries where they live 22. Ensuring clients have immediate access to their results along with their health provider will increase client awareness of the importance of adherence. Peter *et al*. describe the lessons learned from scale‐up of other laboratory tests, such as EID and CD4+ cell count assays, which can inform scale‐up of viral load testing 23. Ellman *et al*. discuss the optimal viral load threshold to use when defining virological failure 24, while Saito *et al*. describe the unique experience of providing viral load results to individuals participating in the PHIA Project 25. Specific issues related to viral load testing among pregnant women, infants and children, adolescents and selected key populations are described in articles by Lesosky *et al*. 26, Arpadi *et al*. 27, Marcus *et al*. 28 and Schwartz *et al*. 29 respectively. Finally, the article by Barnabas *et al*. includes a systematic review of evidence related to the cost‐effectiveness of routine viral load monitoring in low‐ and middle‐income countries 30.

In summary, viral load measurement provides critical information for the management of individuals, as well as insight into the effectiveness of HIV programmes across the entire HIV care continuum. This one test serves as a unique measure of the coverage, quality and impact of HIV programmes. As access to viral load testing expands, it is critical to learn from the lessons of the past, and to take a systems approach to strengthening every step of the viral load continuum 31. All recipients of care deserve access to their viral load test results and all programmes need to move to utilize viral suppression as the indicator of programmatic effectiveness. Ultimately, viral load measurement is an asset that is too precious to waste.

## Competing interests

The authors cite no conflicts of interest.

## Authors’ contributions

All authors contributed equally to the writing and editing of this manuscript.
